# Identification and susceptibility testing of oral candidiasis in advanced cancer patients

**DOI:** 10.1186/s12903-023-02950-y

**Published:** 2023-04-18

**Authors:** Ragnhild Elisabeth Monsen, Anne Karin Kristoffersen, Caryl L. Gay, Bente Brokstad Herlofson, Katrine Gahre Fjeld, Lene Hystad Hove, Hilde Nordgarden, Anita Tollisen, Anners Lerdal, Morten Enersen

**Affiliations:** 1 Department for Interdisciplinary Health Sciences, Institute of Health and Society, Faculty of Medicine, University of Oslo, Postboks 1089 Blindern, 0317 Oslo, Norway; 2 Department of Medicine, Lovisenberg Diaconal Hospital, Oslo, Norway; 3 Institute of Oral Biology, Faculty of Dentistry, University of Oslo, Oslo, Norway; 4 Department of Research, Lovisenberg Diaconal Hospital, Oslo, Norway; 5 Department of Family Health Care Nursing, University of California, San Francisco, USA; 6 Department of Oral Surgery and Oral Medicine, Faculty of Dentistry, University of Oslo, Oslo, Norway; 7 Unit of Oral and Maxillofacial Surgery, Department of Otorhinolaryngology – Head and Neck Surgery Division for Head, Neck and Reconstructive Surgery, Oslo University Hospital, Oslo, Norway; 8 Department of Cariology and Gerodontology, Faculty of Dentistry, University of Oslo, Oslo, Norway; 9 National Resource Centre for Oral Health in Rare Disorders, Lovisenberg Diaconal Hospital, Oslo, Norway; 10 Unger-Vetlesens Institute, Lovisenberg Diaconal Hospital, Oslo, Norway

**Keywords:** Advanced cancer, Fungal, Oral, Etest, Candida carriage, Candidiasis, Polyfungal infection

## Abstract

**Background:**

Patients with advanced cancer are prone to develop different opportunistic oral infection due to anti-cancer treatment or the malignancies themselves. Studies of oral fungal samples show an increased prevalence of non-*Candida albicans* species in mixed oral infections with *Candida albicans*. Non-*C. albicans* and *C. albicans* are associated with varying degrees of resistance to azoles, which may have implications for treatment. This study aimed to assess the diversity and antifungal susceptibility of *Candida* species detected in the oral cavity.

**Methods:**

An observational study with microbiological analysis was conducted. Clinical fungal isolates were collected from patients in a hospice unit in 2014–2016. Isolates were re-grown on chromID® Candida plates in 2020. Single colony of each species was re-cultivated and prepared for biochemical identification with a VITEK2® system and verified by gene sequencing. Etest was performed on RPMI agar, and the antifungals fluconazole, amphotericin B, anidulafungin and nystatin were applied.

**Results:**

Fifty-six isolates from 45 patients were identified. Seven different *Candida* species and one *Saccharomyces* species were detected. The results of biochemical identification were confirmed with sequencing analysis. Thirty-six patients had mono infection, and nine out of 45 patients had 2–3 different species detected. Of *C. albicans* strains, 39 out of 40 were susceptible to fluconazole. Two non-*C. albicans* species were resistant to fluconazole, one to amphotericin B and three to anidulafungin.

**Conclusion:**

*C. albicans* was the predominant species, with a high susceptibility to antifungal agents. Different *Candida* species occur in both mono and mixed infections. Identification and susceptibility testing may therefore lead to more effective treatment and may prevent the development of resistance among patients with advanced cancer.

**Trail registration:**

The study Oral Health in Advanced Cancer was registered at ClinicalTrials.gov (#NCT02067572) in 20/02/2014.

## Background

The oral cavity supports a diverse microbial community that co-exists in symbiosis and equilibrium with the host in healthy individuals. *Candida* species, and predominantly *Candida albicans*, are part of the commensal microbiota in the oral cavity, and normally harmless to the host [1]. In healthy individuals, *C. albicans* accounts for 35% to 55% of the incidence of oral colonization [2]. However, if the immune system is impaired, commensal fungal species can become opportunistic and cause superficial mucosal infections, which may develop into systemic and invasive infections with high morbidity and mortality [3, 4]. Oral mucosal candidiasis commonly presents as a mild infection, but severe and relapsing colonization has been observed in patients receiving antibiotic or corticosteroid treatment [5]. Furthermore, anti-cancer treatment, such as cytotoxic chemotherapy, radiotherapy, drugs or the malignancies themselves, can also make cancer patients prone to different opportunistic oral infections [6, 7]. The prevalence of oral fungal colonization in patients with malignancy has been reported to be more than 70% during and after cancer treatment [6]. Oral fungal infections may lead to local discomfort, thrush/plaque, burning pain, altered taste sensation, poor nutrition, and prolonged hospitalization [8]. In this respect, the symptoms of severe oral candidiasis may strongly impact patients’ quality of life [6].

Several *Candida* species are frequently found in the oral cavity in patients suffering from advanced cancer. According to a number of cohort studies in this patient group, different *Candida* species may have a prevalence of 36%-86% [9–13]. In the same populations, the prevalence of mixed *Candida* infections has been reported to be 10%-47% [9, 10, 13–15].

*C. albicans* is considered to be the most pathogenic of the *Candida* species, whereas *Candida tropicalis*, *Candida glabrata*, *Candida parapsilosis*, *Candida krusei* and others may contribute significantly to morbidity and mortality [16, 17]. The increasing prevalence of non*-C. albicans* species in oral mixed infections may create a more complex clinical picture and have implications for treatment [18], as species such as *C. glabrata* and *C. krusei* harbour inherently less susceptibility to the antifungal agent group of azoles [19]. Insufficient microbiological identification and susceptibility testing of *Candida* species in the oral cavity can result in untreated and recurrent fungal infections, with the potential to spread to esophagus [20] and other parts of the body. This may contribute to increased resistance to antifungal agents.

To address this gap in knowledge in a clinical context, we conducted a microbiological study to extend the understanding of fungal variation and antifungal susceptibility in patients prone to develop oral fungal infections. Therefore, the aims of this study were to explore the diversity of *Candida* species detected in the oral cavity of patients with advanced cancer and to test their susceptibility to available antifungal medications.

## Methods

This was an observational study with a multidisciplinary approach between Lovisenberg Diaconal Hospital and the Faculty of Dentistry, University of Oslo. The study was part of the Oral Health in Advanced Cancer (OralHAC) (NCT02067572) project [21]. Microbiological samples were collected at Lovisenberg Diaconal Hospital between February 2014 and September 2016.

### Patients and setting

Patients in a hospice unit were recruited. Study inclusion criteria were: ≥ 18 years of age and able to provide written informed consent, diagnosis of advanced cancer and patient report of current oral discomfort (i.e., dry mouth, sourness, pain, altered taste). Patients currently or recently treated with antifungal medication were excluded. Patient demographic and medical characteristics were obtained from their medical records.

### Clinical evaluation

Dentists performed an oral examination on each patient, which included an assessment of oral dryness (i.e. sliding mirror test, scored yes/no) [22], clinical signs of fungal infection (i.e. erythema and white plaque on buccal mucosal, scored yes/no), dental plaque scored according to Mucosal-Plaque Score (MPS) [23], white mucosal plaques on the tongue, and registration of the total number of teeth and remaining root remnants and the use of dentures. Oral mucosal inflammation was scored using the Oral Mucositis Assessment Scale (OMAS) [24] (Table 1).

**Table 1:** Patient characteristics (*N* = 45)

Characteristics	Total N	Mean (SD) [min, max] or n (%)
*Sociodemographic variables*		
Age in years	45	65.3 (9.7) [45, 84]
Female sex	45	31 (69)
*Data from medical records*		
Smoking	44	10 (23)
Cancer Diagnosis	45	
Gastro-intestinal		13 (29)
Lung		8 (18)
Gynecologic		7 (16)
Prostate		2 (4)
Breast		2 (4)
Other		13 (29)
Metastases	44	35 (80)
Number of medical treatments	44	12.2 (4.0) [6,26]
Karnofsky score	43	51.2 (18.2) [20, 80]
*Observations and screening by dentists*		
Dental status		
Number of teeth	44	24.7 (4.9) [9,31]
Number of prostheses	42	0.4 (2.0) [0, 13]
Number of tooth remnants	43	0.2 (0.7) [0, 4]
Oral Mucositis (OMAS)		
Ulceration score (range 0–3)	44	0.15 (0.32) [0, 1.67]
Erythema score (range 0–2)	44	0.39 (0.33) [0, 1.11]
Clinical Evaluation		
MPS—plaque on tongue (range 1–4)	43	1.9 (0.9) [1,4]
MPS—plaque on teeth (range 1–4)	44	1.9 (0.9) [1,4]
Clinical sign of dry mouth (yes/no)	40	19 (48)
Clinical sign of fungal infection (yes/no)	43	26 (60)

The 43 excluded patients did not differ from the 45 included patients on any of the variables above

### Oral fungal sampling

Oral swabs sampled from the tongue were immediately inoculated onto chromID® Candida plates (bioMérieux, France) and cultured for 48 h at 37 °C. The chromID® plates from each patient were inspected for the presence of fungal colonies [25], collected and stored at -76 °C. Oral fungal samples from 45 of 88 OralHAC patients were made available for analysis in 2020.

### Biochemical identification

The available samples were re-cultured on a chromID® Candida and incubated aerobically at 37 °C for 72 h. After visual inspection, one colony of each phenotypical type from each sample was selected for further analysis, re-cultured aerobically for 24 h at 37 °C on a Sabouraud Glucose agar (bioMérieux, France), and prepared for biochemical identification by the VITEK®2 (bioMérieux, France) system, according to the manufacturer’s manual.

### DNA sequencing

DNA extractions of fungal colonies were performed by the MasterPure DNA isolation kit from Epicentre (MCD85201, Epicentre Biotechnologies, WI). The internal transcribed spacer (ITS) region between the *18S* and *28S rRNA* genes was amplified with polymerase chain reaction (PCR) using ITS1, 5’TCCGTAGGTGAACCTGCGG’3 forward primer, and ITS4, 5’TCCTCCGCTTATTGATATGC’3, reverse primer [26]. PCR analysis was performed in a 20 µl mixture of OneTaq Mastermix (M048S, New England Biolabs) and DNA template (10–20 ng) in an Applied Biosystem PCR cycler. The reaction includes a denaturation step at 96 °C and 30 amplification cycles: 96 °C for 30 s, 57 °C for 25 s, 72 °C for 30 s. All PCR products were verified by gel electrophoresis and purified using Agencourt Ampure beads (Beckman Coulter). The PCR products were sequenced using BigDye Terminator v1.1 (Applied Biosystems), and ITS4 sequencing primer on ABI 3730 (Applied Biosystems) after being purified with an iX-Pure™ DyeTerminator Cleanup kit from NimaGen (6500 AB Nijmegen, The Netherlands). All sequences were analyzed by Sequencher 5.3 (Gene Codes Corporation, MI) and blasted by NCBI Basic Local Alignment Search Tool (BLAST) [27].

### Susceptibility testing

Antifungal susceptibility testing was performed with an ETEST® (bioMérieux, France) and Ezy MIC™ (HiMedia Laboratories, India). These Etest strips were applied to the inoculated Roswell Park Memorial Institute (RPMI) agar plates (bioMérieux, France) and incubated at 37 °C. Each isolate was tested against four different antifungal agents: fluconazole (FL) 0.016–256 µg/ml, amphotericin B (AB) 0.002–32 µg/ml, anidulafungin (AND) 0.002–32 µg/ml, and nystatin Ezy MIC™ strip (NYT) 0.002–32 µg/ml. The minimum inhibitory concentration (MIC) of the antifungal agents was visually measured for each Etest strip after 48 h. The MIC registration in each analysis was correlated according to the breakpoint recommendations by the European Committee on Antimicrobial Susceptibility Testing (EUCAST) [28].

Nystatin susceptibility was also supplemented with a disk diffusion test, where sterile paper disks were soaked in a 20 µl Nystatin Orifarm 100 000 IU/ml mixture, ordered from the Lovisenberg Diaconal Hospital pharmacy. The concentration of 20 µl of nystatin 100 000 IU/ml corresponds to 2000 IU on each disk diffusion [29]. According to the Regional Medicine Information and Pharmacovigilance Center in Norway (RELIS), nystatin 100 000 IU/ml is compatible with 20 000 µg/ml (20 µg/µl), and 20 µl with 2000 IU corresponds to approximately 400 µg nystatin on each disk diffusion [30]. The inhibition zone diameter for nystatin disk diffusion was measured after 48 h with a digital caliper 0–150 mm. For nystatin, the supplementary susceptibility testing was categorized according to the inhibition zone diameters from the Clinical and Laboratory Standards Institutes: ≥ 15 mm = susceptible; 14–10 mm = susceptible dose-dependent; and < 10 mm = resistance [31, 32].

## Results

Of the 88 patients in the OralHAC Study, 45 had oral fungal swabs available for analysis and were included in this study. Demographic and medical data for the 45 included patients are summarized in Table 1. Clinical evaluation indicated that 19 (48%) of the patients had clinical signs of dry mouth, and 26 (60%) had clinical signs of oral fungal infection. The OMAS mean scores were 0.15 and 0.39 for ulceration and erythema, respectively. The 45 included patients did not differ from the 43 excluded patients on any measured demographic or medical characteristics.

### Identification

#### *Biochemical identification*

**Fig. 1:**
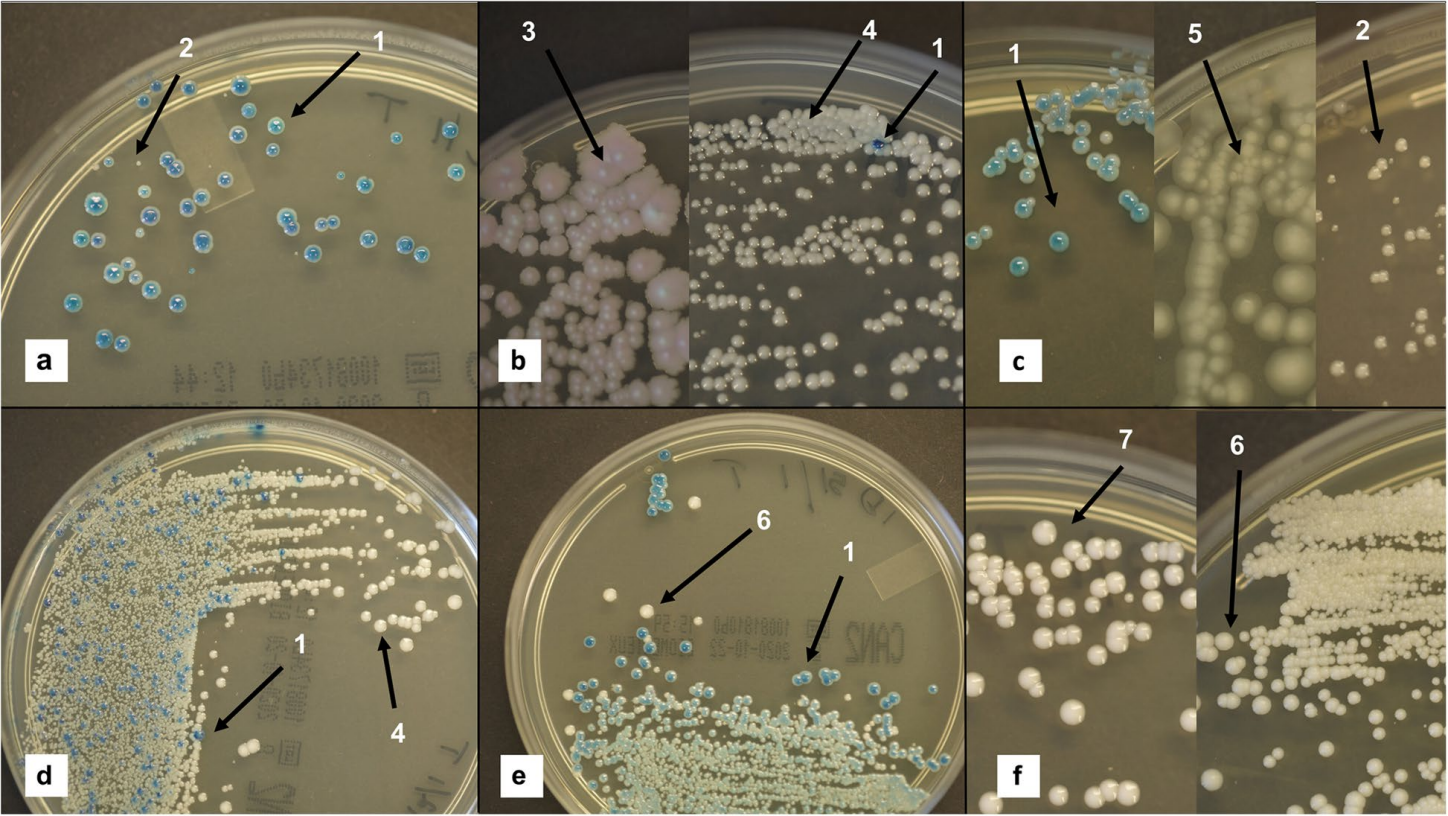
Photos of polyfungal infections grown on chromID® Candida from nine patients, and identified visually. Each picture (**a**—**f**) represents the different combinations of species. Picture **a)** One patient with the combination *C. albicans* (1/blue-green) and *Saccharomyces cerevisiae* (2/white); **b**) One patient with *C. tropicalis* (3/pink), *C. glabrata* (4/white) and *C. albicans* (1/ blue-green); **c)** One patient with *C. albicans* (1/blue-green), *C. krusei* (5/white, flat, irregular) and *Saccharomyces cerevisiae* (2/white, convex, regular); **d)** Three patients with *C. albicans* (1/blue-green) and *C. glabrata* (4/white); **e)** Two patients with *C. albicans* (1/blue-green) and *C. parapsilosis* (6/white); **f)** One patient with *C. guilliermondii* (7/white-slightly pink) and *C. parapsilosis* (6/white)

Samples from 45 patients resulted in 56 detected isolates. Seven different *Candida* species and one *Saccharomyces* species were identified. In the 56 isolates, *C. albicans* comprised 71%, *C. glabrata* 9%, *C. parapsilosis* 5%, and *C. tropicalis* 4%. Furthermore, *C. krusei*, *Candida guilliermondii* and *Candida dubliniensis* accounted for 2% each, and *Saccharomyces cerevisiae* for 5%. In 36 patients, only one species was detected, with *C. albicans* accounting for 32 (89%). Colonies of each species, *C. glabrata*, *C. tropicalis*, *C. dubliniensis* and *Saccharomyces cerevisiae* were detected in four different patients, respectively. A summary is presented in Table 2. In nine patients, two or three different species were found. The combination of C*. albicans* and *C. glabrata* was most frequent, and was registered in four of nine patients (Fig. 1). The results of the VITEK®2 identifications had a ‘confidence of probability’ that ranged from “Good” to “Excellent”.

**Table 2:** Detected oral fungal species in 45 patients, and the number of species distributed in the group of 56 isolates

Species	Number (%) of patients *N* = 45	Number (%) of patients with mono colony *N* = 36	Number (%) of isolates *N* = 56
*C. albicans*	40 (89)	32 (89)	40 (71)
*C. glabrata*	5 (11)	1 (3)	5 (9)
*C. parapsilosis*	3 (7)	0	3 (5)
*C. tropicalis*	2 (4)	1 (3)	2 (4)
*C. krusei*	1 (2)	0	1 (2)
*C. guillermondii*	1 (2)	0	1 (2)
*C. dubliniensis*	1 (2)	1(3)	1 (2)
*Saccharomyces cerevisiae*	3 (7)	1(3)	3 (5)

#### *Genetic identification*

The biochemical identification was confirmed for all 56 isolates by gene sequencing of the interspace region between *18* and *28S rRNA* with the universal ITS1 + ITS4 primers [26].

### Susceptibility testing

#### *Etest*

*C. albicans* was susceptible to fluconazole (MIC ≤ 4 µg/ml) in 39 of 40 isolates (98%), with only one isolate resistant to fluconazole (MIC of 6 µg/ml). Furthermore, *C. albicans* was susceptible to both amphotericin B and anidulafungin in all 40 isolates. Four of five *C. glabrata* were susceptible to fluconazole with MIC values of 0.5, 1.5, 2 and 16 µg/ml (Table 3). The one *C. glabrata* resistant to fluconazole had a MIC value of 24 µg/ml.

**Table 3:** Susceptibility of antifungal agents on *Candida* isolates

Species	Number isolates *N* = 56	Fluconazole 0.016–256 µm/ml		Amphotericin B 0.002–32 µg/ml		Anidulafungin 0.002–32 µg/ml	
		S	R	S	R	S	R
		n (%)	n (%)	n (%)	n (%)	n (%)	n (%)
*C. albicans*	40	39 (98)	1 (2)	40 (100)	0	40 (100)	0
*C. glabrata*	5	4^a^ (80)	1 (20)	5 (100)	0	5 (100)	0
*C. parapsilosis*	3	3 (100)	0	3 (100)	0	1 (33)	2 (67)
*C. tropicalis*	2	2 (100)	0	1 (50)	1(50)	1 (50)	1 (50)
*C. krusei*	1	-	-	1 (100)	0	1 (100)	0
*C. guilliermondii*^b^	1	IE	IE	IE	IE	IE	IE
*C. dubliniensis*^c^	1	1 (100)	0	1 (100)	0	NR	NR
*Saccharomyces cerevisiae*^d^	3	ND	ND	ND	ND	ND	ND

*S* Susceptible, *R* Resistant

^a^Notes from EUCAST: “The entire *C. glabrata* is in the Intermediate category. MICs against *C. glabrata* should be interpreted as resistant when above 16 mg/L. Susceptible category (≤ 0.001 mg/L) is simply to avoid misclassification of "I" strains as "S" strains”

^b^IE Insufficient evidence that the organism or group is a good target for therapy with the agent Fluconazole according to EUCAST

^c^EUCAST: Anidulafungin not recommended for this species

^d^EUCAST: Not determined

All isolates tested, except one *C. tropicalis* isolate (MIC of 1.5 µg/ml), were susceptible to amphotericin B. Two *C. parapsilosis* (MIC of 6 and > 32 µg/ml) and one *C. tropicalis* (MIC of 0.125 µg/ml) were resistant to anidulafungin. The third *C. parapsilosis* had a MIC of 4 µg/ml, just below the threshold for resistance.

The *C. albicans* isolate with the fluconazole MIC value of 4 µg/ml, which is close to resistance (MIC > 4 µg/ml), occurred in combination with *C. glabrata* (MIC fluconazole < 2 µg/ml). In another patient with the same combination, both species *C. albicans* and *C. glabrata* were susceptible to fluconazole. The patient with the fluconazole resistant *C. albicans* (MIC of 6 µg/ml) was also detected in combination with *C. glabrata* and *C. tropicalis.* Furthermore, susceptibility was present in this patient for fluconazole in C. *glabrata* (MIC of 2 µg/ml) and *C. tropicalis* (MIC of 0.5 µg/ml), while *C. tropicalis* was resistant to amphotericin B (MIC of 1.5 µg/ml) (Table 3).

#### *Disk diffusion test*

**Fig. 2:**
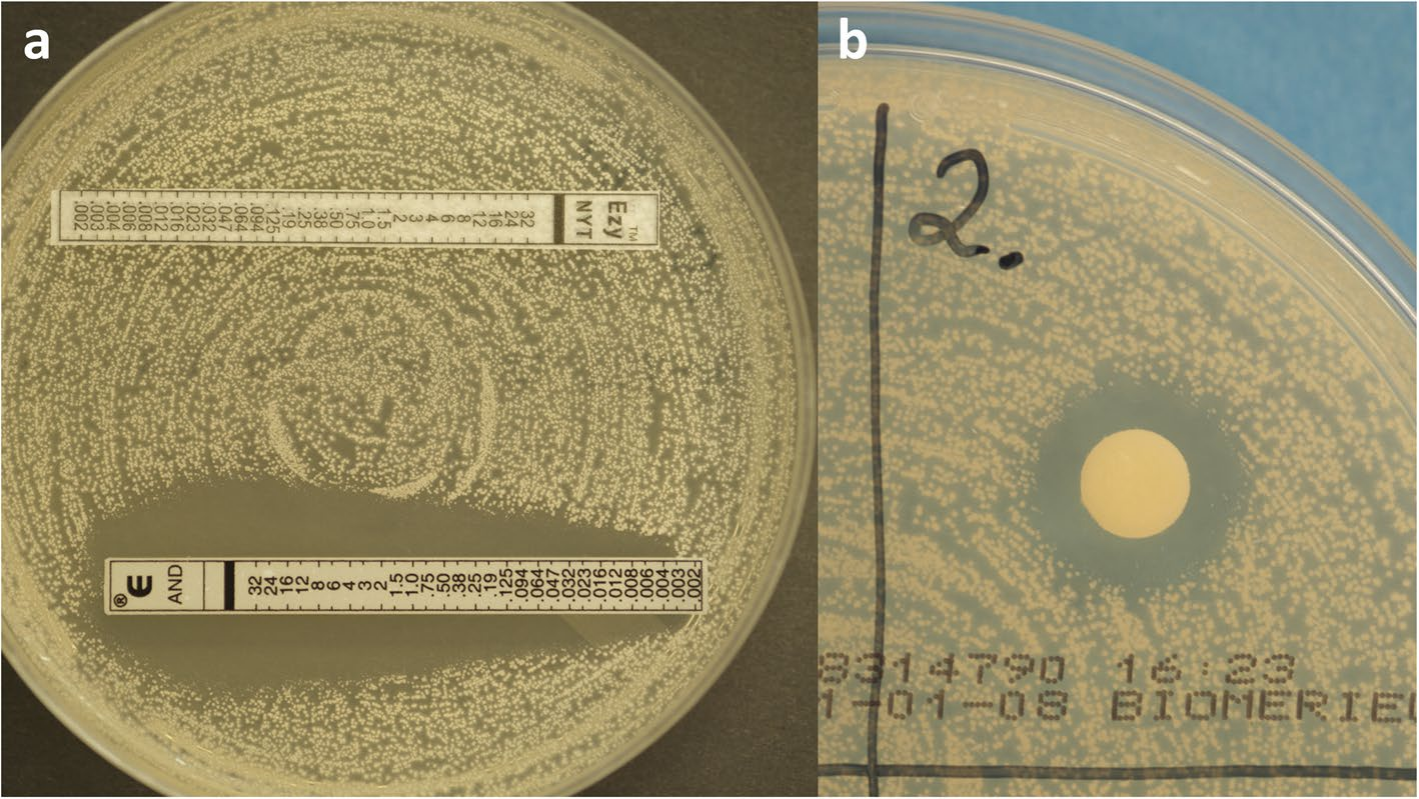
Photos of susceptibility testing of nystatin Etest and paper disk, with suspension on RPMI agar plates. Picture **a**) Etest anidulafungin (AND) 0.002-32µg/ml with clear inhibition zone and Etest nystatin Ezy MIC™ strip 0.002-32 µg/ml with no inhibition zone. Picture **b**) A supplementary test with nystatin mixture 100 000 IU/ml, 20 µl applied to sterile paper disks. All 30 isolates had clear inhibition zones

When using Etest for nystatin (Ezy MIC™) with a concentration range 0.002–32 µg/ml, no inhibition zone appeared for any of the *Candida* isolates, and the results are therefore not included in Table 3. Due to these insufficient results, we had to conduct a supplementary test with nystatin mixture 100 000 IU/ml (20 µl) by disk diffusion for those 30 isolates that were available at that time. The results verified a clear inhibition zone (Fig. 2). Twenty-six isolates had values in the intermediate range (I = 14–10 mm), and three *C. albicans* and one *C. glabrata* had a resistance profile (R < 10 mm) (Table 4).

**Table 4:** Supplementary analysis of susceptibility using paper disks with nystatin mixture 100 000 IU/ml

Species	Number of isolates *n* = 30	Nystatin mix 100 000 IU/ml (20 µl) Sterile paper disk 48 h		
		S (S > 15 mm) n (%)	I (I = 14–10 mm) n (%)	R (R < 10 mm) n (%)
*C. albicans*	23	0	20 (87)	3 (13)
*C. glabrata*	3	0	2 (67)	1 (33)
*C. parapsilosis*	2	0	2 (100)	0
*C. tropicalis*	1	0	1 (100)	0
*C. krusei*	0	0	0	0
*C. guillermondii*	0	0	0	0
*C. dubliniensis*	1	0	1 (100)	0
*Saccharomyces cerevisiae*	0	0	0	0

*S* Susceptible, *I* Intermediate, *R* Resistance

## Discussion

The present study was part of the OralHAC project and investigated the diversity of oral yeasts in patients with advanced cancer. Few studies include microbiological identification and susceptibility testing of samples from oral infections in this patient group [33]. The microbiological analysis in our study verified oral fungal cultures in all 45 patients, and 56 fungal isolates were collected. However, 19 (40%) of the patients did not have clinical signs of fungal infection (i.e., white confluent plaques, bleeding surfaces, erythematous or ulcerated fissures) [34]. Additionally, the OMAS scores showed a low burden of erythema and ulceration, and could support the asymptomatic feature that oral fungal infections may express.

Among the 56 isolates, *C. albicans* dominated with a prevalence of 71%. This number is higher than reported in other studies of advanced cancer patients with fungal infection, where the prevalence of *C. albicans* was reported to be 43%—56% [9, 10, 14, 35]. In our study, two or three different species were detected in 20% of the patients. This number is slightly lower than prior studies where the same number of species were detected in 23% to 47% [9, 10, 14, 15]. *C. albicans* with *C. glabrata* is the most common combination of species, detected in approximately 70% of patients with oral candidiasis [18, 31]. In the current study, this combination occurred in only 44% of patients. This variation in results across studies may be explained by epidemiological factors, differing methods of species identification, and variation in sample size and patient characteristics, including previous antifungal treatment [19, 36].

Mixed infection with *C. albicans* and non-*C. albicans* may lead to more complex infections, which could result in poor treatment response, as well as drug resistance [37]. *C. albicans’* ability to change from yeast form to the state of hyphae formation makes it highly pathological with host tissue invasion and local infection as a result [37]. The morphological switch is controlled by the host’s immune system, and makes patients with advanced cancer prone to *C. albicans* proliferation [38]. In their mouse study, Tati et al. (2016) described how *C. albicans* formation of hyphae create conditions for *C. glabrata´s* co-adherence to the hyphae, and spread of the infection to oropharyngeal candidiasis. Furthermore, the authors describe how different *C. albicans* stocks, with inconsistent susceptibility to the azole agents, may affect *C. glabrata’s* susceptibility to the same agents [39]. Though we cannot compare their study to our results, due to different methodologies, the study by Tati et al. highlights the complexity of polyfungal infections. Although *C. glabrata* is inherently less susceptible to the azoles, we observed susceptibility to fluconazole in three *C. glabrata* isolates in combination with *C. albicans,* with MIC values ranging 0.5–2 µg/ml. It is worth noticing that, according to EUCAST, these values are considered to be in the intermediate category (MIC > 0.001–16 µg/ml), and therefore dose-dependent for therapeutic success.

In the present study, 98% of *C. albicans* isolates were susceptible to fluconazole. This result corresponds with a study from Denmark, where no acquired azole resistance was detected for *C. albicans* [10]. There was only one resistant *C. albicans* isolate in this study (fluconazole MIC of 6 µg/ml), and it was detected in a mixed combination with *C. glabrata* and *C. tropicalis*. In the same patient, the *C. tropicalis* isolate was resistant to amphotericin B. *C. tropicalis* is not frequently detected in mucosal infections, and it is only able to invade the oral epithelium in hyphae/pseudo-hyphae form [18]. However, when *C. tropicalis* enters the blood stream, it is considered to be a significant pathogen in patients with neutropenia and malignancies [19], with a high predictive value for invasiveness in neutropenic patients [6]. In the other patient with *C. tropicalis,* the isolate was a mono colony, susceptible to amphotericin B.

Three *C. parapsilosis* species were detected in this study. Two isolates, both in combination with *C. albicans*, were resistant to anidulafungin. *C. parapsilosis* is known to be intrinsically less susceptible to the echinocandins [18, 19, 40], but is far less virulent and poorly invasive compared to *C. albicans*. Nevertheless, *C. parapsilosis* may induce significant tissue damage, related to specific protein expression when growing in contact with the epithelium [18].

There was no observed susceptibility to the tested concentrations of nystatin in the Etest in this study. Therefore, we performed a supplementary analysis with nystatin mixture used in clinical treatment. The mixture concentration was ten times higher than the nystatin Etest. A clear susceptibility zone was observed and measured for all 30 isolates (Fig. 2). Most of the fungal isolates were found to be in the intermediate group, where there is a likelihood of therapeutic success by adjusting the dose regime. However, topical treatment with polyene agents, such as nystatin and amphotericin B, is not recommended in the Norwegian guidelines for palliative care in cancer, based on little evidence of success. The recommendation is for antifungal agent fluconazole [41], although nystatin mixture agents are frequently prescribed to manage uncomplicated oral candidiasis [5]. Resistance to polyenes by *Candida* is rare [17], and insignificant resistance to the polyenes is also reported in studies in cancer patients [32, 42]. However, in one study, almost half of the isolates were detected to be in the intermediate sensitivity group [42]. The nystatin concentrations used in this study (nystatin dilution 0.125–64 µg/ml and disk diffusion 50 µg) were low compared to our supplementary analysis with the nystatin mixture.

*Candida* species are extremely heterogeneous, both pheno- and genotypically, and the pathogenicity of each species is mediated by virulence factors such as adhesins, morphological switching, biofilm formation and secretion of enzymes to invade and damage the epithelium [43]. The feature of virulence and susceptibility in the *Candida* organisms, in combination with changes in the host’s status, may impact the clinical outcome of antifungal treatment [19]. Oral fungal treatment in patients with advanced cancer is often prescribed without confirmation of species and antifungal susceptibility [44]. In immunocompromised patients, this may cause severe and recurrent fungal infections and increase the levels of distressing symptoms and reduce quality of life.

### Limitations and future directions’

A limitation of this study was the small patient sample size that prevents us from comparing clinical subjective and objective features with the microbiological results. Our study was only descriptive and outlines features of oral yeasts in patients with advanced cancer. A future prospective study, with an expanded sample size and incorporating comparisons of microbiological diagnoses with clinical subjective and objective symptoms, could yield further knowledge for establishing guidelines for diagnostics and treatment of oral fungal infections in this patient group.

## Conclusion

This study showed that different *Candida* species may occur in both mono and mixed yeast infections. *C. albicans* was the predominant species, with a high susceptibility to antifungal agents. However, identifying polyfungal infections is important, as they may contribute to treatment failure. Microbiological sampling and analysis with regard to identification and susceptibility testing need to be considered as a supplementary diagnostic option for some patients with advanced cancer.

## Data Availability

The dataset generated and analyzed during the current study is stored on Lovisenberg Diaconal Hospital’s research server, in accordance with Norwegian ethical and legal requirements. Oral fungal sample tests are stored at the Institute of Oral Biology, University of Oslo. Requests for access to an anonymized minimal data set can be sent to the corresponding author. Minimal anonymized data set can be shared, but only after approval from the Data Protection Officer at Lovisenberg Diaconal Hospital and the Regional Committees for Medical and Health Research. The microbiological datasets generated and analysed during the current study are available in the National Library of Medicine, National Center for Biotechnology Information, accession number (OQ099739-OQ099794), https://www.ncbi.nlm.nih.gov/nucleotide/.

## Abbreviations

OralHAC: Oral Health in Advanced Cancer
MPS: Mucosal-Plaque Score
OMAS: Oral Mucositis Assessment Scale
ITS: Internal Transcribed Spacer
PCR: Polymerase Chain Reaction
RPMI: Roswell Park Memorial Institute
FL: Fluconazole
AB: Amphotericin B
AND: Anidulafungin
NYT: Nystatin Ezy MIC™ strip
MIC: Minimum Inhibitory Concentration
EUCAST: European Committee on Antimicrobial Susceptibility Testing

## References

[1] Andersen KM, Kristoffersen AK, Ingebretsen A (2016). Diversity and antifungal susceptibility of Norwegian Candida glabrata clinical isolates. J Oral Microbiol.

[2] Marsh PD, Lewis MAO, Rogers H, Wilson M (2016). Oral Microbiology.

[3] Niimi M, Firth NA, Cannon RD (2010). Antifungal drug resistance of oral fungi. Odontology.

[4] Arastehfar A, Gabaldón T, Garcia-Rubio R, et al. Drug-Resistant Fungi: An Emerging Challenge Threatening Our Limited Antifungal Armamentarium. Antibiotics (Basel). 2020;9(12). 10.3390/antibiotics9120877. 10.3390/antibiotics9120877 PMC7764418 33302565

[5] Quindós G, Gil-Alonso S, Marcos-Arias C (2019). Therapeutic tools for oral candidiasis: current and new antifungal drugs. Med Oral Patol Oral Cir Bucal.

[6] Lalla RV, Latortue MC, Hong CH (2010). A systematic review of oral fungal infections in patients receiving cancer therapy. Support Care Cancer.

[7] Chitapanarux I, Wongsrita S, Sripan P (2021). An underestimated pitfall of oral candidiasis in head and neck cancer patients undergoing radiotherapy: an observation study. BMC Oral Health.

[8] Akpan A, Morgan R (2002). Oral candidiasis. Postgrad Med J.

[9] Davies A, Brailsford S, Broadley K, Beighton D (2002). Oral yeast carriage in patients with advanced cancer. Oral Microbiol Immunol.

[10] Astvad K, Johansen HK, Høiby N, Steptoe P, Ishøy T (2015). Oropharyngeal candidiasis in palliative care patients in Denmark. J Palliat Med.

[11] Fischer D, Epstein J, Yao Y, Wilkie D (2014). Oral health conditions affect functional and social activities of terminally ill cancer patients. Support Care Cancer.

[12] Wilberg P, Hjermstad MJ, Ottesen S, Herlofson BB (2012). Oral health is an important issue in end-of-life cancer care. Support Care Cancer.

[13] Alt-Epping B, Nejad RK, Jung K, Groß U, Nauck F (2012). Symptoms of the oral cavity and their association with local microbiological and clinical findings—a prospective survey in palliative care. Support Care Cancer.

[14] Bagg J, Sweeney M, Lewis MA (2003). High prevalence of non-albicans yeasts and detection of anti-fungal resistance in the oral flora of patients with advanced cancer. Palliat Med.

[15] Davies AN, Brailsford SR, Beighton D, Shorthose K, Stevens VC (2008). Oral candidosis in community-based patients with advanced cancer. J Pain Symptom Manage.

[16] Richardson JP, Moyes DL, Ho J, Naglik JR (2019). Candida innate immunity at the mucosa. Semin Cell Dev Biol.

[17] Lewis MAO, Williams DW (2017). Diagnosis and management of oral candidosis. Br Dent J.

[18] Silva S, Negri M, Henriques M (2012). Candida glabrata, Candida parapsilosis and Candida tropicalis: biology, epidemiology, pathogenicity and antifungal resistance. FEMS Microbiol Rev.

[19] Arendrup MC (2013). Candida and candidaemia. Susceptibility Epidemiology Dan Med J.

[20] Laudenbach JM, Epstein JB (2009). Treatment strategies for oropharyngeal candidiasis. Expert Opin Pharmacother.

[21] Monsen RE, Herlofson BB, Gay C (2021). A mouth rinse based on a tea solution of Salvia officinalis for oral discomfort in palliative cancer care: a randomized controlled trial. Support Care Cancer.

[22] Henricsson V, Svensson A, Olsson H, Axéll T (1990). Evaluation of a new device for measuring oral mucosal surface friction. Eur J Oral Sci.

[23] Henriksen BM, Ambjørnsen E, Axéll TE (1999). Evaluation of a mucosal-plaque index (MPS) designed to assess oral care in groups of elderly. Spec Care Dentist.

[24] Sonis ST, Eilers JP, Epstein JB (1999). Validation of a new scoring system for the assessment of clinical trial research of oral mucositis induced by radiation or chemotherapy. Cancer.

[25] Odds FC, Bernaerts R (1994). CHROMagar Candida, a new differential isolation medium for presumptive identification of clinically important Candida species. J Clin Microbiol.

[26] Raja HA, Miller AN, Pearce CJ, Oberlies NH (2017). Fungal Identification Using Molecular Tools: A Primer for the Natural Products Research Community. J Nat Prod.

[27] Altschul SF, Gish W, Miller W, Myers EW, Lipman DJ (1990). Basic local alignment search tool. J Mol Biol.

[28] The European Committee on Antimcrobial Susceptibility Testing Subcommittee on antifungal Susceptitibility Testing (EUCAST-AFST). Breakpoint tables for interpretation of MICs for antifungal agents 2020 [updated 2020–02–04 April 2021]. Version 10.0: Available from: https://www.eucast.org/astoffungi/clinicalbreakpointsforantifungals/. Accessed 28 Sept 2021.

[29] Nenoff P, Krüger C, Neumeister C, Schwantes U, Koch D (2016). In vitro susceptibility testing of yeasts to nystatin–low minimum inhibitory concentrations suggest no indication of in vitro resistance of Candida albicans, Candida species or non-Candida yeast species to nystatin. Clin Med Investig.

[30] RELIS South-East. RELIS South-East. Available from: https://www.relis.no/about_relis/. Accessed 18 Nov 2020.

[31] Miranda-Cadena K, Marcos-Arias C, Mateo E (2018). Prevalence and antifungal susceptibility profiles of Candida glabrata, Candida parapsilosis and their close-related species in oral candidiasis. Arch Oral Biol.

[32] Schelenz S, Abdallah S, Gray G (2011). Epidemiology of oral yeast colonization and infection in patients with hematological malignancies, head neck and solid tumors. J Oral Pathol Med.

[33] Dhaliwal JS, Murang ZR, Ramasamy DTR, Venkatasalu MR (2020). Oral microbiological evidence among palliative patients: an integrated systematic review. Indian J Palliat Care.

[34] Patil S, Rao RS, Majumdar B, Anil S (2015). Clinical Appearance of Oral Candida Infection and Therapeutic Strategies. Front Microbiol.

[35] Ball K, Sweeney MP, Baxter WP, Bagg J (1998). Fluconazole sensitivities of Candida species isolated from the mouths of terminally ill cancer patients. Am J Hosp Palliat Care.

[36] Baumgardner DJ (2019). Oral fungal microbiota: to thrush and beyond. J Patient Cent Res Rev.

[37] Zhou Y, Cheng L, Lei YL, Ren B, Zhou X. The Interactions Between Candida albicans and Mucosal Immunity. Front Microbiol. 2021;12:652725. doi.org/10.3389/fmicb.2021.652725. 10.3389/fmicb.2021.652725 PMC8255368 34234752

[38] Vila T, Sultan AS, Montelongo-Jauregui D, Jabra-Rizk MA. Oral Candidiasis: A Disease of Opportunity. J Fungi (Basel). 2020;6(1). 10.3390/jof6010015. 10.3390/jof6010015 PMC7151112 31963180

[39] Tati S, Davidow P, McCall A (2016). Candida glabrata binding to candida albicans hyphae enables its development in oropharyngeal candidiasis. PLoS Pathog.

[40] Turner SA, Butler G (2014). The Candida pathogenic species complex. Cold Spring Harb Perspect Med.

[41] The Norwegian Directorate of Health. The Norwegian guidelines for palliative care in cancer care. Oslo, Norway: The Norwegian Directorate of Health; 10/2019. https://www.helsedirektoratet.no/retningslinjer/palliasjon-i-kreftomsorgen-handlingsprogram. Accessed Dec 2021.

[42] Davies A, Brailsford S, Broadley K, Beighton D (2002). Resistance amongst yeasts isolated from the oral cavities of patients with advanced cancer. Palliat Med.

[43] Scorzoni L, Fuchs BB, Junqueira JC, Mylonakis E (2021). Current and promising pharmacotherapeutic options for candidiasis. Expert Opin Pharmacother.

[44] Bagg J, Sweeney MP, Davies AN, Jackson MS, Brailsford S (2005). Voriconazole susceptibility of yeasts isolated from the mouths of patients with advanced cancer. J Med Microbiol.

